# SYNTHESIZING LIFESTYLE MEDICINE AND SUCCESSFUL AGING: A PERFECT PAIRING FOR THEORIZING OPTIMAL AGING?

**DOI:** 10.1093/geroni/igae098.3540

**Published:** 2024-12-31

**Authors:** Teri Undem, Heather Fuller

**Affiliations:** North Dakota State University, Fargo, North Dakota, United States; North Dakota State University, Fargo, North Dakota, United States

## Abstract

Within gerontology, various theoretical perspectives on what it means to age successfully have emerged over recent decades (e.g. Rowe & Kahn, etc.) and garnered empirical support. Within medicine and related healthcare fields, research is prolific regarding engaging in healthy living in terms of physical health, mental health, and well-being. In recent years, a theoretical perspective of Lifestyle Medicine has emerged to describe the role of such lifestyle factors on health. The present work includes a scoping review of both literatures with the ultimate goal of determining overlaps and distinctions between theoretical concepts of successful aging and lifestyle medicine. We take a holistic approach to develop a model that identifies linkages between the principles of successful aging and lifestyle medicine. The resultant ‘Lifestyle Model of Optimal Aging’ merges the core pillars of Lifestyle Medicine (nutrition, physical activity, stress management, restorative sleep, social connection, and avoidance of risky substances) with the core principles of successful aging from the models put forth by Rowe and Kahn (1997) and expanded upon by Urtamo et.al., (2019). This new, holistic approach to exploring the linkages between the principles of LM and the essential components of successful aging highlights how proactive behavioral health strategies related to nutrition, exercise, sleep, stress, and substances can foster optimal aging in cognitive and physical functioning and disease prevention, thereby potentially yielding more positive outcomes for aging well. Discussion explores how concepts underlying successful aging may be enhanced by incorporating a lifestyle medicine approach.

